# Hyperferritinemia: Important Differentials for the Rheumatologists

**DOI:** 10.7759/cureus.68588

**Published:** 2024-09-03

**Authors:** Mandeep Kaur, Samantha W.S. Lo, Yixin Liu, Kevin Yip

**Affiliations:** 1 Internal Medicine, Wyckoff Heights Medical Center, New York, USA; 2 Internal Medicine, Icahn School of Medicine at Mount Sinai, Elmhurst Hospital Center, Queens, USA; 3 Social Services, Mount Sinai Health System, New York, USA; 4 Rheumatology, Wyckoff Heights Medical Center, New York, USA

**Keywords:** autoimmune diseases, macrophage activation syndrome (mas), hemophagocytic lymphohistiocytosis (hlh), adult onset still's disease (aosd), hyperferritinemia

## Abstract

Ferritin is commonly used as a marker for iron status, aiding in diagnosing iron deficiency anemia. However, it is also an acute phase reactant often elevated in various inflammatory conditions. Marked hyperferritinemia, defined as ferritin levels above 10,000 μg/L, can indicate severe underlying conditions, including infections, cardiovascular like heart failure, endocrinological, autoimmune, and malignancies. This case report highlights the differential diagnoses and clinical implications of hyperferritinemia from a rheumatological perspective. Here are two case reports illustrating the use of ferritin in aiding the diagnosing of two uncommon conditions: adult-onset Still's disease (AOSD) and hemophagocytic lymphohistiocytosis (HLH). The first case involves a 37-year-old male who presented with a pruritic rash, flu-like symptoms, joint pain, fever, and chills. Despite multiple emergency department (ED) visits, his hyperferritinemia reached 88,000 μg/L, and he met the Yamaguchi criteria for AOSD. Treatment with pulse-dose steroids led to a rapid resolution of symptoms. In the second case, a 50-year-old female presented with sepsis due to recurrent axillary skin infections, needing transfer to the intensive care unit. Laboratory findings revealed hyperferritinemia of 39,671 μg/L, crucial for distinguishing between rheumatological and hematological causes. Further investigation revealed diffuse large B-cell lymphoma. Tragically, the patient succumbed to her illness. The cases highlight the critical role of ferritin as a marker for underlying severe conditions. The clinical interpretation of ferritin levels and appropriate diagnostic workup are essential in identifying and managing these conditions to reduce morbidity and mortality. Ferritin levels should not be overlooked as merely an indicator of iron status or inflammation. Marked hyperferritinemia requires thorough investigation to differentiate between potential underlying conditions that may allow for more prompt recognition and management to reduce morbidity and mortality.

## Introduction

Ferritin levels are commonly used as a marker for iron status and can aid in diagnosing iron deficiency anemia. However, importantly, ferritin is an acute phase reactant and is often elevated in various inflammatory conditions. Inflammatory cascades, such as cytokine release and oxidative stress, increase ferritin secretion [1]. It modifies immunological responses by binding to T and B lymphocytes, modulating delayed hypersensitivity reactions, antibody production, and phagocytosis by neutrophils [2]. Hyperferritinemia is often referred to as markedly elevated ferritin levels above 10,000 μg/L [3]. Such high levels are commonly linked to many cardiovascular and endocrinological disorders, chronic inflammation, multisystem inflammatory syndrome in children (MIS-C) related to COVID-19, autoimmune diseases like macrophage activation syndrome (MAS) and adult-onset Still’s disease (AOSD), as well as malignancies like hemophagocytic lymphohistiocytosis (HLH) [3,4]. This highlights the importance of ferritin as a marker to assess the mortality risk of these diseases, as higher levels could be correlated to severe forms of diseases [5,6].

The etiologies of marked hyperferritinemia are not clearly understood, and HLH and MAS should be considered high differentials in critically ill patients with elevated ferritin levels. Hematological malignancies can contribute to hyperferritinemia themselves while also triggering HLH, underscoring the importance of keeping these rare disorders in mind to avoid missing these life-threatening conditions [7]. HLH can be primary, genetic in origin with defective T cytotoxic lymphocyte function, or secondary, associated with environmental triggers in individuals with some form of genetic alteration, resulting in uncontrolled immune system activation. MAS is a form of secondary HLH linked to rheumatological diseases [8].

Many rheumatological diseases, such as rheumatoid arthritis, AOSD, Kawasaki disease, juvenile idiopathic arthritis, or SLE flare, can trigger inflammatory cascades triggering MAS [9]. MAS and HLH can be clinically characterized by end-organ damage, disseminated coagulopathy, depression in all cell lines, and inappropriate activation of macrophages in the setting of marked inflammation, resulting in phagocytosis of blood cells, termed hemophagocytosis [8]. Apart from rheumatological conditions, hyperferritinemia also aids in the diagnosis of secondary HLH caused by infections such as dengue virus, and this complication is associated with a high mortality rate [10]. With all these clinical outcomes, marked hyperferritinemia reflects the extent of the underlying cytokine storm. Patients often end up in critical care from multiorgan failure, and 25% of patients lose their lives, outlining the gravity of these disorders. Higher mortality rates were observed in malignancy-associated HLH compared to rheumatological diseases in a multiple cause-of-death analysis done in France [10,11]. Ferritin is commonly ordered as part of the primary workup for patients, but often, the results are not utilized to their best potential as clinical interpretation may seem complex. A lack of appropriate approach toward high ferritin results and disregard for its potential as a non-specific marker hinders obtaining a conclusive etiology. In our paper, we are discussing two rare hyperferritinemia-associated disorders, comparing hematological malignancy versus rheumatological origin of inflammation with entirely different outcomes.

## Case presentation

Case report 1

**Figure 1:**
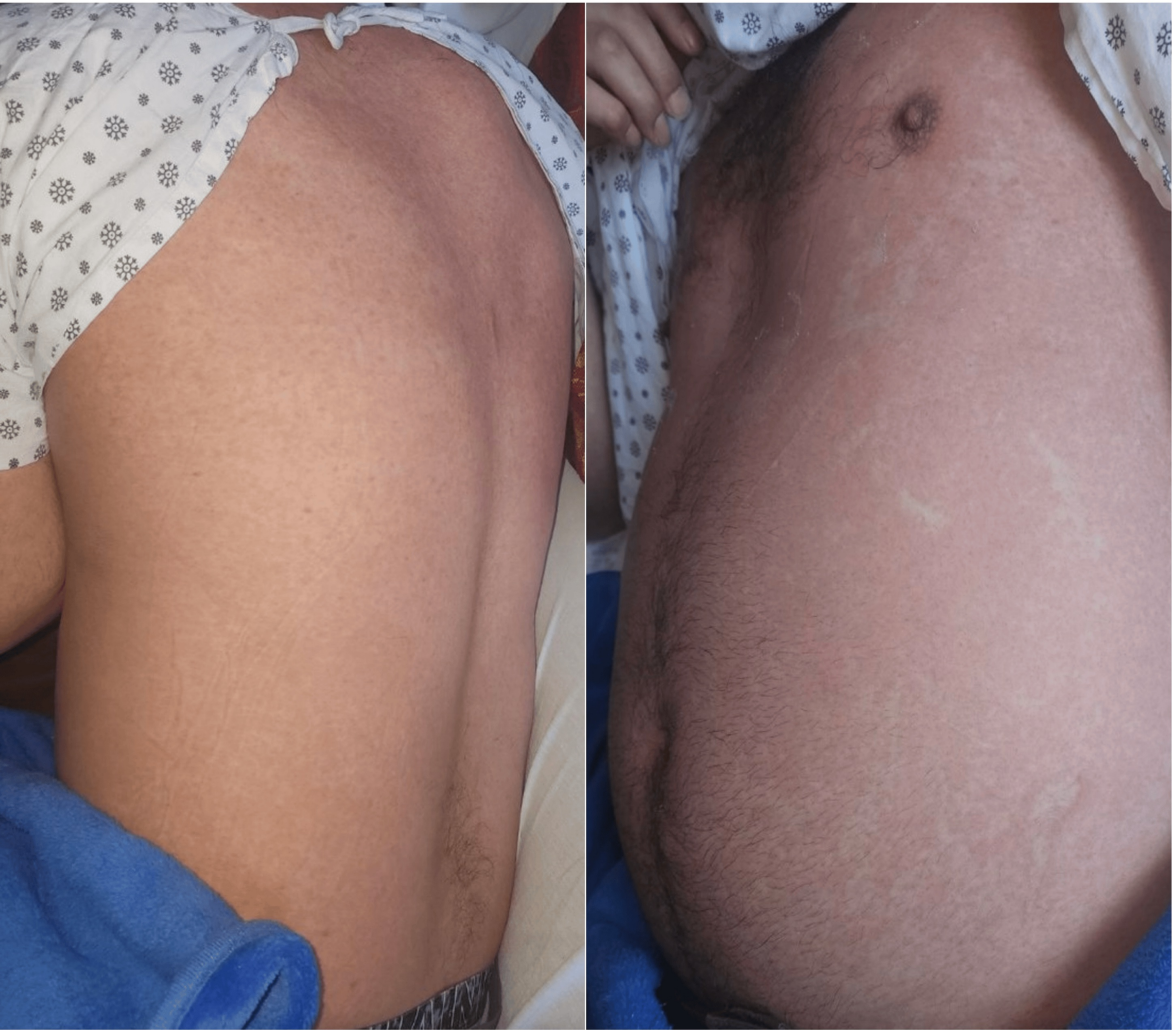
Salmon-colored rash on the chest and back

Our first case is a 37-year-old Hispanic male with no known past medical history. He was initially evaluated in the emergency department (ED) for a pruritic rash on his upper back and left lower abdomen. The patient was discharged from the ED with antihistamines for a localized allergic reaction. He later returned to the ED with complaints of flu-like symptoms, intense joint pain, intermittent subjective fever, chills, and a persistent rash on his chest and back (Figure 1).

Laboratory blood work was significant for a positive throat swab for group A *Streptococcus* (GAS), leukocytosis at 11,000, AST at 43, and ALT at 87. A chest X-ray was negative for acute pathology. The patient was treated with clindamycin 900 mg IV and discharged with a diagnosis of GAS throat infection, prescribed oral clindamycin 300 mg three times a day for 10 days. A few days later, the patient returned to the ED with a persistent but worsening sore throat, muscle aches, joint stiffness, and intermittent fever. Labs showed leukocytosis of 19,900, AST at 58, and ALT at 149. HIV antigen-antibody was nonreactive, but there was lactic acidosis of 4.8, CRP at 232, and ESR at 96. The patient received an intramuscular penicillin G injection of 1.2 million units and a stat dose of vancomycin 1500 mg. A CT neck with IV contrast was normal, and the patient was discharged the same day. It was recommended that the patient follow up with an outpatient physician. The patient returned to the ED with a worsening rash on his body, denying any sick contacts. He was admitted to the medicine service with suspicion of acute rheumatic fever secondary to GAS. On the fourth visit, admission labs were significant for persistent leukocytosis and worsening AST at 460 and ALT at 847. An ultrasound of the abdomen showed a fatty liver with a nonreactive hepatitis panel for hepatitis A, B, and C. During the initial days of admission, the patient complained of migratory arthralgia in the bilateral wrists, right shoulder, right knee, and left ankle, with redness and swelling of affected joints. X-ray imaging of the affected joints showed no acute pathology. Antistreptolysin O and anti-DNase B titers were within normal ranges. Naproxen was started, and after appropriate NSAID dosing, the arthralgia resolved.

Although the patient had symptomatic relief, he continued to have a persistent rash and worsening liver function. He was found to have hyperferritinemia of 88,000 and an IL-2 level of 13,277. The patient met the Yamaguchi criteria for AOSD, including arthralgia for more than two weeks, typical rash, WBC greater than 10,000, sore throat, and abnormal liver enzymes, with GAS being the suspected triggering factor. Following the diagnosis of AOSD, the patient received three days of methylprednisolone 1000 mg, resulting in quick resolution of symptoms, followed by a slow tapering of high-dose steroids during his inpatient stay. Fever episodes resolved, with down-trending ferritin levels, CRP, ESR, and improving AST/ALT levels (Table 1). The patient was discharged on oral steroids. In the outpatient setting, the patient was found to have a resolving rash on physical examination and a further downtrend in inflammatory markers, with overall clinical improvement. The patient returned to the community without long-lasting sequelae.

**Table 1:** Laboratory values for case 1 WBC, white blood count; GAS, group A *Streptococcus*; ESR, erythrocyte sedimentation rate; CRP, C-reactive protein; AST, aspartate transaminase; ALT, alanine transaminase; IL-2, interleukin-2

Parameter	Inpatient stay	Post-discharge
WBC (4000-11,000/µL)	19.9	10.2
GAS swab	Detected	NA
ESR (0-15 mm/h)	96	35
CRP (<1 mg/L)	232	35
Ferritin (24-336 ng/L)	92,999	1568
AST (8-48 IU/L)	158	15
ALT (7-55 IU/L)	308	37
IL-2 (0-31.2 pg/mL)	13,277	NA
Anti streptolysin AB (<166 Todd units)	128	NA
Anti-DNase B (0-302 U/mL)	192	NA

Case report 2

Our second case involves a 50-year-old African American female Jehovah’s Witness with a past medical history of hypertension, diabetes mellitus, depression, and hidradenitis suppurativa. She was admitted for sepsis secondary to a recurrent deep-seated skin infection in the axillary region complicated by hospital-acquired pneumonia. Before this admission, she had been treated for an axillary infection with intravenous antibiotics at another hospital. Upon physical examination, the patient was alert and oriented, with an open wound in the left axillary region of 5 cm in size and pitting bilateral lower extremity edema. A CT scan of her left upper extremity revealed an open wound with prominent lymph nodes in the left axilla but no abscess. Initially, she was admitted to the medical ward and treated with multiple broad-spectrum antibiotics. However, her hospital course was complicated by pancytopenia, recurrent fevers, widespread lymphadenopathy, and worsening mentation, leading to her transfer to the ICU. Her workup revealed significantly elevated ferritin levels of 39,671 ng/mL; LDH, 2,352 U/L; ALP, 571 U/L; AST, 330 U/L; ALT, 72 U/L; D-dimer, 7,170.16 ng/mL; and fibrinogen, 282 mg/dL (Table 2). HIV Ag/Ab was negative, ESR was 64 mm/h, CRP was 139 mg/L, and IL-2 receptor was 14,315 pg/mL. The marked hyperferritinemia prompted a differentiation between underlying rheumatological and hematological etiologies. A rheumatological cause for the severe inflammatory process was ruled out due to the absence of related signs and symptoms, as well as a negative autoantibody panel, which included C-ANCA, myeloperoxidase antibody, cyclic citrullinated peptide IgG, mixed connective tissue disease, SLE, and Sjogren’s disease antibody panel. A lymph node biopsy showed diffuse large B-cell lymphoma (DLBCL). However, due to the presence of sepsis and increasing bilirubin levels, chemotherapy and radiation therapy were not initiated. For this patient, the calculated H score for reactive hemophagocytic syndrome was 253 points, with a probability of more than 99%. The underlying malignancy in this case suggested a hematologically triggered HLH, although a bone marrow biopsy was negative. It is worth noting that the patient was receiving steroid treatment, which could have affected the biopsy results. Unfortunately, the patient expired in critical care, highlighting a significantly different outcome than our first case.

**Table 2:** Laboratory values for case 2 WBC, white blood count; ESR, erythrocyte sedimentation rate; CRP, C-reactive protein; LDH, lactate dehydrogenase; ALP, alkaline phosphate; AST, aspartate transaminase; ALT, alanine transaminase; IL-2, interleukin-2

Parameter	Inpatient stay
WBC (4000-11,000/µL)	1630
ESR (0-20 mm/h)	64
CRP (<1 mg/L)	134
LDH (135-214 U/L)	2352
Fibrinogen (200-400 mg/dL)	282
D-dimer (0-0.5 mg/L)	7170
Ferritin (24-336 ng/L)	39,671
ALP (44-147 IU/L)	571
AST (10-36 IU/L)	330
ALT (5-38 IU/L)	72
IL-2 (0-31.2 pg/mL)	14,315

## Discussion

**Figure 2:**
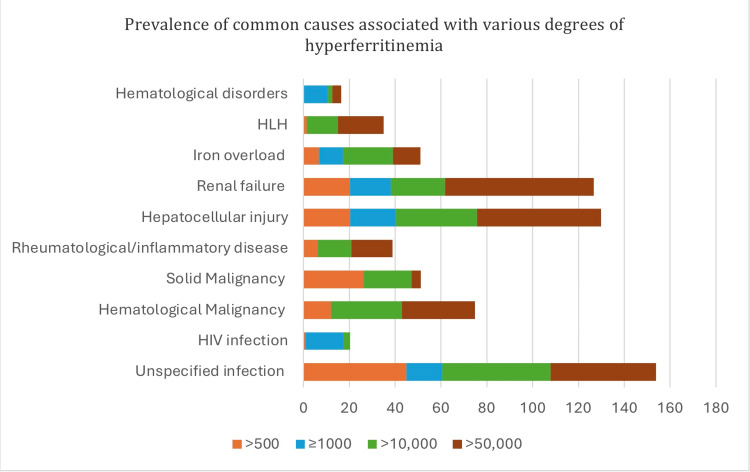
Prevalence of common causes associated with various degrees of hyperferritinemia The X-axis shows ferritin levels (μg/L), and the Y-axis shows various disease entities.

Ferritin levels can uncover much greater and grave diseases in the shadows of just being representatives of iron overload. It is important to consider a broader differential diagnosis of high ferritin levels in clinical practice. Marked hyperferritinemia is labeled as ferritin levels above 10,000, which was seen in both of our cases. Sackett et al. studied ferritin levels in more than 60,000 cases, out of which only less than 1% showed marked hyperferritinemia, similar to the study by Crook and Walker that demonstrated similar results [12,13], further supporting our clinical thought of how rare marked hyperferritinemia can be. In 2023, La Marle et al. published a high-scale multiple cause-of-death analysis, which outlayed etiologies of hyperferritinemia and linked the prevalence of high ferritin levels in common versus rare disorders [11]. Figure 2 demonstrates that high ferritin levels can be seen in very common disorders like iron overload, infections, and renal failure; however, it also marks that there are few diseases with poor prognosis that a clinician cannot afford to miss [1].

There is limited literature investigating associations of hyperferritinemia with rheumatological diseases. Orbach et al. reported that SLE, rheumatoid arthritis, and dermatomyositis were associated with hyperferritinemia in 23%, 4%, and 15% of cases, respectively [5]. In contrast, a small tertiary care rheumatology center study found that rheumatological conditions accounted for approximately 60% of hyperferritinemia cases, with AOSD, RA, and SLE being standard differentials [7]. Hematological malignancies contribute to 3% of cases of marked hyperferritinemia, a higher proportion than solid malignancies. High ferritin levels are not specific to HLH but correlate with increased prevalence of the condition [14]. Clinical evaluation and prognostication are crucial alongside laboratory findings in establishing a diagnosis. Ferritin levels can be used in monitoring disease response to treatment, as seen in the first case, where appropriate treatment with steroids led to improvement in ferritin levels. Undiagnosed underlying rare disorders can lead to higher mortality, as evidenced by our reported cases. In our second case, the patient required ICU admission and did not survive. Critically ill patients with HLH have an approximate 60% mortality rate, worsened by the severity of inflammation and organ dysfunction [15]. In 2018, Gars et al. conducted a study comparing the yields of bone marrow biopsies in patients with HLH and those without HLH [16]. They concluded that significant hemophagocytosis does not reliably predict HLH without clinical features suggestive of the disease. However, substantial hemophagocytosis remains a relatively uncommon finding. While the presence of hemophagocytosis may aid clinicians in diagnosing HLH, its absence does not exclude the diagnosis. Clinical correlation remains paramount, as demonstrated in our second case, where a bone marrow biopsy yielded negative results [16]. Markedly raised serum ferritin level is strongly associated with HLH, and a cutoff value of >10000 mcg/L was 90% sensitive and 96% specific [17].

## Conclusions

Ferritin levels, often perceived merely as an inflammatory marker, can be elevated in conditions such as unspecified infections, iron overload, and renal failure. However, it is crucial to recognize and thoroughly investigate the differentials of marked hyperferritinemia. Conditions such as AOSD, HLH, and MAS, regardless of their underlying etiology, are associated with high mortality rates. Identifying and managing these conditions by allocating appropriate resources to interpret hyperferritinemia can be life-saving. Accurate diagnosis and timely intervention are essential in reducing the morbidity and mortality associated with these severe conditions.
